# UPLC-ESI-QTOF-MS Profiling of Phenolic Compounds from *Eriocephalus africanus*: In Vitro Antioxidant, Antidiabetic, and Anti-Inflammatory Potentials

**DOI:** 10.3390/molecules27248912

**Published:** 2022-12-15

**Authors:** Kunle Okaiyeto, Nasifu Kerebba, Oluwafemi O. Oguntibeju

**Affiliations:** 1 Phytomedicine and Phytochemistry Group, Department of Biomedical Sciences, Faculty of Health and Wellness Sciences, Cape Peninsula University of Technology, Bellville 7535, South Africa; 2 Department of Chemistry, Makerere University, Kampala P.O. Box 7062, Uganda

**Keywords:** *Eriocephalus africanus*, antioxidant, antidiabetic, anti-inflammatory, phytochemicals, phenolic compounds, UPLC-ESI-QTOF-MS

## Abstract

The present study investigated phenolic compounds, antioxidant, antidiabetic, and the anti-inflammatory potentials of methanolic and chloroform extracts of *Eriocephalus africanus*. The methanolic extract included, polyphenols (112 ± 2.81 mg gallic acid equivalent (GAE)/g), flavonols (76.12 ± 7.95 mg quercetin equivalents (QE)/g); antioxidant capacity (Ferric Reducing Antioxidant Power (FRAP) (752.64 ± 89.0 μmol of ascorbic acid equivalents (AAE) per g dry weight (µmol AAE/g), 2,2-dyphenyl-1-picrylhydrazyl (DPPH) (812.18 ± 51.12 Trolox equivalents per gram of dry mass of plant extracts (μmol TE/g), TEAC (631.63 ± 17.42 µmol TE/g)), while the chloroform extract included polyphenols (39.93 ± 1.36 mg GAE/g), flavonols (44.81 ± 3.74 mg QE/g); antioxidant capacity, DPPH (58.70 ± 5.18 µmol TE/g), TEAC (118.63 ± 3.74 µmol TE/g) and FRAP (107.10 ± 2.41 µmol AAE/g). The phytochemicals profiling performed by UPLC-ESI-QTOF-MS revealed some important polyphenols, predominantly flavonoids, that could be responsible for the antioxidant capacity and biological effects. Both extracts demonstrated a dose-dependent manner of the alpha-glucosidase inhibition with an IC_50_ between 125 and 250 μg/mL for methanolic extract, while the chloroform extract was at 250 μg/mL. In the L6 myoblasts and C3A hepatocytes, the methanolic extract slightly increased the utilization of glucose, and both extracts exhibited a dose-dependent increase in the glucose uptake in both cell types without significantly increasing the cytotoxicity. Furthermore, both extracts exhibited an anti-inflammatory potential and the findings from the present study could serve as a baseline for further research in the development of pharmaceutical agents.

## 1. Introduction

The number of people with metabolic diseases, such as diabetes and obesity, is increasing across the globe [1]. According to a 2017 estimate from the International Diabetes Federation, 451 million individuals globally have diabetes, and by 2045, there will probably be 693 million diabetic cases [2]. The excessive consumption of carbohydrates and triglycerides is one of the main factors that contribute to the metabolic syndrome [3]. It is relatively common and affects people all over the world who consume too much energy and do not exercise regularly. Type 2 diabetes (T2D), which is one of these metabolic syndromes, and is defined by hyperglycaemia and the impaired carbohydrate metabolism, it is a substantial economic burden and a leading cause of morbidity and death, globally [4,5]. The majority (>95%) of newly diagnosed diabetics have type 2 diabetes (T2D), which is driven by the pancreatic beta cell failure and insulin resistance [6]. Obesity is the cause of insulin resistance and pancreatic beta-cell death, which establishes the connection between T2D and obesity [7]. The onset and progression of T2D complications are significantly influenced by postprandial blood glucose levels [8]. There is a connection between diabetes and the immune defense system, and it is known that this association deteriorates in the chronic diabetic conditions, causing significant injury to the relevant tissues and organs [9].

A physiological condition, known as oxidative stress, occurs when the levels of reactive oxygen species (ROS) and reactive nitrogen species (RNS) increase abnormally, either because of an excessive generation or inadequate clearance when the antioxidant system of an individual has been overpowered by the effect of the reactive species [10]. These very reactive chemicals, which are by-products of the regular cellular metabolism, are essential components of most signaling pathways. One of the key factors contributing to the development of diabetes is oxidative stress [11]. As previously reported by Al-Aubaidy and Jelinek [12], the connection between oxidative stress, inflammation, and type 2 diabetes, reducing oxidative stress and inflammation is essential for advancing the healing process and preventing diabetic complications. The relationship between inflammation and oxidative stress is thought to contribute to the development of chronic diseases [4]. According to the pathophysiology of type 2 diabetes, oxidative stress is one of the factors that contributes to the pathogenesis of insulin resistance, impaired insulin secretion, impaired glucose uptake, impaired hepatic glucose metabolism, combined with activation of the inflammation of pro-inflammation cytokines, and ultimately type 2 diabetes [13,14]. It is established that elevated glucose levels (hyperglycaemia) and inflammatory reactions cause oxidative stress in pancreatic beta cells [15]. The aetiology of diabetes is facilitated by the overpowering of the antioxidant system by oxidants, which is why diabetic individuals have a higher oxidative status than healthy subjects, i.e., a greater amount of ROS generation [16]. As a result of the increased oxidative damage to the essential macromolecules, several studies have demonstrated a direct connection between oxidative stress and diabetes [4,17].

The medicinal use of antioxidants found in naturally occurring phytochemical substances for removing reactive species, has increased because of the increased awareness of their role in reducing the effects of oxidative stress in diabetic conditions [18]. In diabetic conditions, oxidative stress leads to insulin resistance, beta cell malfunction, and insulin secretion, all of which may be influenced by the phytochemicals with strong antioxidant properties [19,20]. Inhibiting α-amylase and α-glucosidase is one of the therapeutic methods for treating postprandial hyperglycaemia [21]. Due to the numerous adverse effects, these enzymes relate to conventional antidiabetic drugs, such as acarbose and miglitol, which are frequently regarded as ineffective, for the management and treatment of type 2 diabetes (T2DM), despite the enormous advances made in their development [22].

Due to their minimal to no side effects, the study of medicinal plants and natural products, as therapeutic sources for the treatment of T2DM, has been gaining popularity over the years [23]. It has been demonstrated that bioactive compounds derived from natural sources can reduce blood sugar levels in a variety of ways [1,18,24]. Typically, plants contain a variety of bioactive substances, including several targets for diabetes treatment [20]. According to several studies, phytochemicals, such as phenolics, have inhibitory effects on α-amylase and α-glucosidase, and may be used therapeutically to treat diabetes [1,21,25]. Medicinal plants that strengthen the body’s antioxidant system, lower the blood pressure, maintain a low blood sugar, and regulate insulin, are safer options for the treatment of T2D [22,26].

The native South African plant *Eriocephalus africanus* (Asteraceae), sometimes known as wild rosemary, has naturalized throughout the Mediterranean and is widely used in traditional medicine [27]. Due to the widespread distribution in the veld and its thin, grey leaves that, when crushed, release the Vicks fragrance, it is one of the shrubs that most Cape inhabitants are familiar with. Furthermore, it is a well-known medicinal plant and a great shrub for the waterwise garden [28]. It has a very broad range of distribution, especially when compared to plants that thrive in the salty coastal air and others that grow in more dry inland environments. They all grow into silvery, grey, bushy, evergreen shrubs, up to one m in height, in general. In the Western Cape, Eastern Cape, and Namaqualand regions of South Africa, *E. africanus* is mostly distributed on clay and granite slopes. Wild rosemary has long been used as a remedy for a variety of illnesses, including colds and coughs, flatulence, and colic. It is also used as a diuretic and a diaphoretic [28]. Several studies have reported on the therapeutic benefits of *E. africanus*, including its antibacterial [29], anticancer [30], and antioxidant properties [27,31].

There is a dearth of information on the phytochemical compounds and pharmacological uses of *E. africanus*, despite the growing number of health benefits that folk medicine believes this plant possesses. In general, the plant is still under-utilized and therefore, the present study investigated the antioxidant, antidiabetic, and anti-inflammatory potentials of *E. africanus* in vitro, with the hope that this study will stand as a reference source for further research.

## 2. Results and Discussion

### 2.1. Phytochemical Compounds and Antioxidant Activity

Diabetes mellitus is one of the most prevalent endocrine diseases and poses a serious threat to global public health. The search for a safer option has become imperative because the conventional drugs used to manage diabetes are associated with side effects. Remarkably, natural antioxidant-rich products containing phenolic compounds have gained attention for the treatment of oxidative stress-related diseases. The most prevalent secondary metabolites identified in plants and widely spread throughout the plant kingdom are phenolics [32]. Plant secondary metabolites serve many functions, including those related to innate immunity [33], defense response signaling [34], and plant growth and development processes [35]. Interestingly, the potential health advantages of plant polyphenolics have been reported in various studies [36,37,38]. Plant phenolics have antioxidant properties that have been scientifically demonstrated to prevent a variety of chronic diseases and oxidative stress-related illnesses [39,40]. In the present study, both polar (70% methanol) and non-polar solvents (chloroform) were used as the extraction solvents. From 100 g of the plant powder extracted with 1 L of the extraction solvents, 22.4 g and 15 g yields were obtained from the methanol and chloroform extracts, respectively.

The polyphenol and flavonol contents in the crude methanolic and chloroform extracts of *E. africanus* were investigated and the results are represented in Table 1. It was observed that the polyphenol content (112 ± 2.81 mg GAE/g) in the methanolic extract of *E. africanus* was higher, as compared to the chloroform extract (39.93 ± 1.36 mg GAE/g). Similarly, the flavonol content of the methanolic extract of *E. africanus* was higher (76.12 ± 7.95 mg QE/g), when compared with that of the chloroform extract (44.81 ± 3.74 mg QE/g). Concomitantly, the antioxidant capacity of the extracts was also investigated, and it was recorded that the methanolic extract has a higher antioxidant capacity (FRAP—752.64 ± 89.0 µmol AAE/g; DPPH—812.18 ± 51.12 µmol TE/g; TEAC—631.63 ± 17.42 µmol TE/g), as compared to the chloroform extract (FRAP—107.10 ± 2.41 µmol AAE/g; DPPH—58.70 ± 5.18 µmol TE/g; TEAC—118.63 ± 3.74 µmol TE/g). The higher antioxidant capacity of the methanolic extract, as compared to the chloroform extract could be due to the high contents of polyphenols and flavonols. The higher amount of both polyphenols and flavonols in the methanolic extract, compared to the chloroform extract, is an indication of the possible therapeutic role of plants and plant-based products [32,41].

Different phenolic compounds may exhibit different solubility characteristics, due to their varied chemical structures, which range from simple to polymerized forms. Medium polar and polar phenolic substances, such as phenolic acids and flavonoid glycosides, are frequently extracted using methanol [42]. The extraction solvent has a significant effect on the nature of the phytochemical compounds that will be extracted. The solvent polarity influences the extraction of the phenolic compounds from medicinal plants, and this could be responsible for the higher phenolic compounds and antioxidant capacity of the methanolic extract of *E. africanus* over the chloroform extract [32]. The higher antioxidant potential of the methanolic extract is mostly due to the nature of the phenolic compounds [43,44].

### 2.2. Phytochemical Analysis of the Methanol and Chloroform Extracts of E. africanus

**Figure 1:**
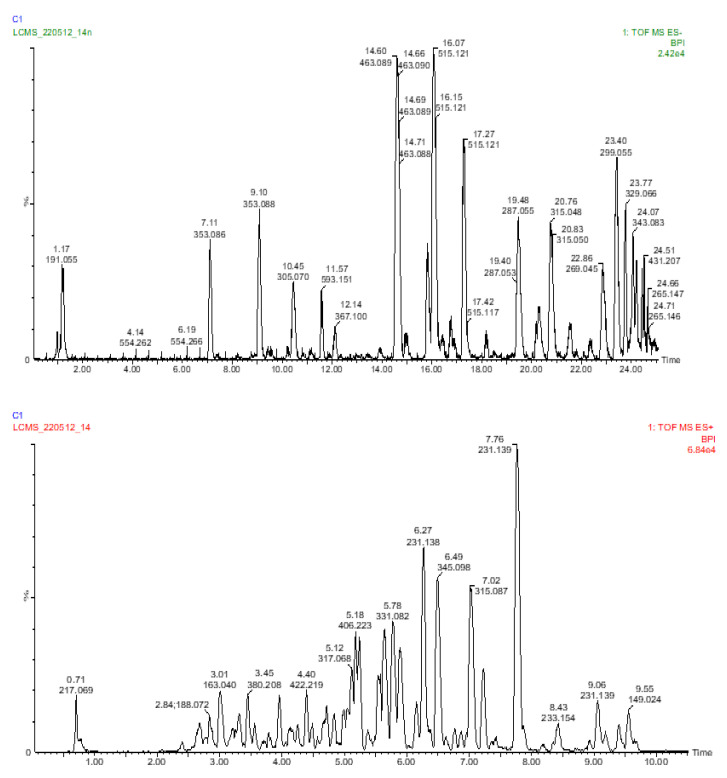
UHPLC-ESI-MS base peak chromatogram for the methanol extract of *E. africanus*’s negative and positive ion modes.

**Figure 2:**
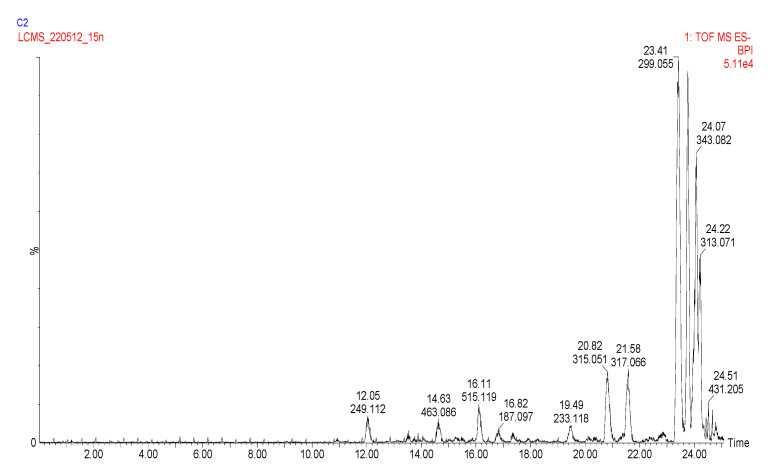

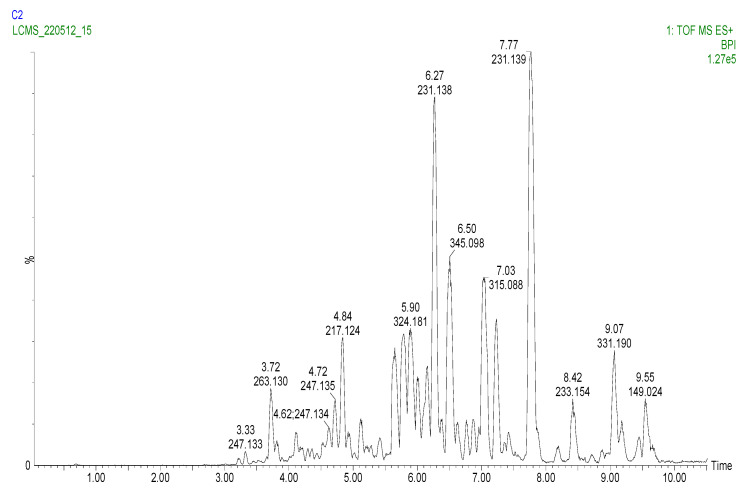
UHPLC-ESI-MS base peak chromatogram for the chloroform extract of *E. africanus*’s negative and positive ion modes.

**Figure 3:**
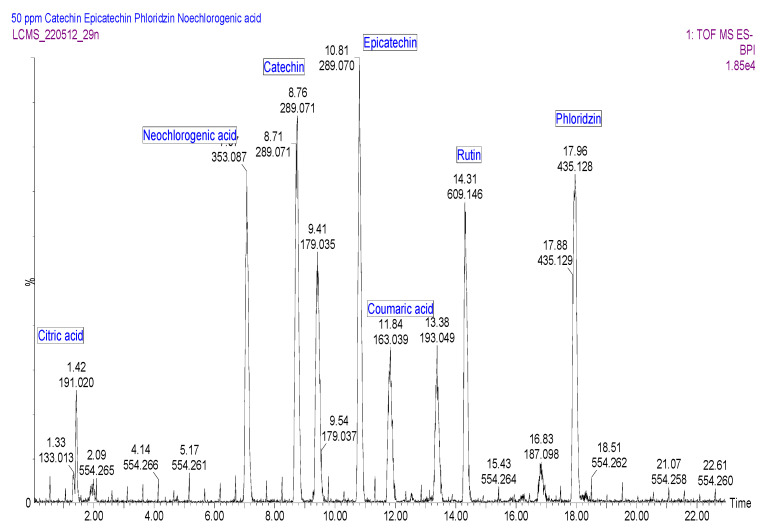
UHPLC-ESI-MS base peak chromatogram for the standard mix in the negative ion mode.

Sixty-two compounds were identified in *E. africanus* methanol and chloroform extracts, through a high-resolution UHPLC-QTOF-MS analysis (Table 2 and Table 3, Figure 1 and Figure 2). The compounds were identified using their full mass spectra and MS/MS fragmentation patterns, and then compared to the published data or MS/MS databases, such as Metlin, MassBank, PubChem, etc. In addition, a standard mixture (Figure 3) was used to completely identify metabolites, using their retention time and fragmentation. The standard mixture was prepared by injecting 50 ppm of each of the chemical marker compounds. A total of 48 metabolites were identified from the methanol extract, compared to 28 metabolites identified from the chloroform extract of *E. africanus*.

### 2.3. Analysis of the Phenolic Compounds and Other Metabolites

In total, 62 metabolites could be identified from the methanol and chloroform extracts of *E. africanus*. Flavonoids were the most identified compounds among which flavones and flavonols were the most represented. Other classes of organic compounds included carboxylic acids, hydroxicinnamic acid, flavan-3-ols, and coumaric and furanocoumaric compounds.

#### 2.3.1. Analysis of the Carboxylic Acids

Two carboxylic acid peaks could be identified, i.e., peaks 7 and 36. The fragmentation of the carboxylic acids occurred through the release of one or two water (18 Da) and carbon dioxide (44 Da) molecules or both (62 Da), and is characterized by the ion fragments [M − H-18]^−^ and CO_2_, [M − H-44]^−^, or [M − H-62]^−^. Peak 7 was identified as gemfibrozil with *m/z* [M − H]^−^, 249 and *m/z* 205 [M − H-44]^−^, 231 [M − H-18]^−^, 187 [M − H-62]^−^. Peak 36 was identified to be trans-3-indoleacrylic acid, an acid of an alkaloid nature using other data. The compound showed a [M + H]^+^ ion at *m/z* 188. The MS^2^ fragment at *m/z* 146 was due to [M + H-CO_2_]^+^.

#### 2.3.2. Analysis of the Hydroxycinnamic Acids

In the present study, nine hydroxycinnamic acids or derivatives were tentatively or completely identified (peaks 1, 2, 3, 4, 8, 12, 13, 15, and 16). Six quinic acid (QA) derivatives were identified, including two earlier reported: caffeoyl quinic acid (CQA), from this plant (peaks 3 and 4) [27], and di-CQAs (12, 13, 15 and 16). Their spectra showed a deprotonated molecular ion at *m/z* 353 of mono-CQAs and *m/z* 515 of di-CQA. The fragmentation in the MS/MS produced *m/z* 191 (QA), which gave a dehydrated quinic acid moiety (*m/z* 173) and caffeic acid (*m/z* 179), as a prominent fragment. The 3-O-CQA (neochlorogenic acid) that eluted at RT 7.11 min and 1-O-CQA (chlorogenic acid) (UV maxima at 326 nm) were differentiated by the intensity of the characteristic ions of the chlorogenic acids and the retention time comparison with that of the standard. In the former, *m/z* 179 is the base peak and in the latter, *m/z* 191 is the base peak [46,47]. For di-O-CQAs, the more the additional caffeoyl groups attached to the less free equatorial hydroxyl groups (owing to steric interactions) retained in the quinic acid residue, the stronger the retention [48]. That is, the loss of the caffeoyl group (C) is likely to be in the order; 1 − C > 5 − C > 4 − C > 3 − C [46,48]. This enabled peaks 12, 13, 15, and 16 to be identified as 1,5 di-O-CQA, 3,4 di-O-CQA, 3,5 di-O-CQA and 1,4 di-O-CQA, respectively, since the elution order is 1,5-diCQA << 3,4-diCQA << 3,5-diCQA << 1,4-diCQA [46]. The prominent ions for 3,5-di-CQA have *m/z* 179 as base peaks, consistent with earlier studies [47,49,50]. *E. africanus* has also earlier been reported to be one of the sources of 3,4 di-O-CQA, 3,5 di-O-CQA, and 1,4 di-O-CQA [27]. Peak 8 was identified as that of feruloyl quinic acid having [M − H]^–^ ion at *m/z* 367 and MS^2^ fragments *m/z* 191 [quinic – H]^–^, 173 [quinic acid − H-H_2_O]^–^, 134 [Feruloyl-CO_2_-CH_3_]^–^. Another hydroxycinnamic acid derivative was identified in peak 1 as p–coumaric acid ethyl ester, having shown a deprotonated ion at *m/z* 191 with the MS^2^ characteristic ion of the coumaroyl at *m/z* 119 [coumaric acid-CO_2_]^–^. An acylated derivative of caffeic acid, a caffeoyl–hexuronide derivative was identified with adduct [M − H + Cl]^–^, *m/z* 554 with fragments *m/z* 355 (after the loss of 162 Da, probably that of the caffeoyl moiety or of a hexose) and then of a hexuronide acid (*m/z* 113), as earlier reported from this plant [27].

#### 2.3.3. Analysis of the Flavanones, Flavones, Flavonols, and Flavan-3-ols

Flavanones, flavones, flavonols, and flavan-3-ols were the most identified flavonoids. The retro-Diels–Alder was used to study the flavonoid fragmentation pathways (RDA) and 14 flavones were identified. Peaks 6, 27, 32, and 33 were identified to belong to that apigenin or its C-linked sugar conjugate, as earlier reported [45], or newly reported from this plant using the fragmentation pattern in earlier reports. Peaks 14, 21, 22, 27, 28, 29, 30, 31, 32, 33, 51, 55, and 56 could show ion peaks at *m/z* 284 or 285 [M − H]^–^ and 287 [M + H]^+^, corresponding to that of luteolin, and thus identified as its derivative. This was supported by the UV maximum absorption for the flavones. Peaks 29 and 51 were identified as that of tricin in both positive and negative ion modes with [M + H]^+^ *m/z* 331 and [M − H]^−^; *m/z* 329, respectively. The MS^2^ fragment *m/z* 315 was due to the loss of methyl. Following the flavones, 12 flavonols were also identified. They showed UV maxima at around 346 nm. Peaks 11, 17, 23, 24, 25, 45, 46, 47, 48, 50, 52, and 53 were flavonol peaks. Peak 11 and 46 with MS or MS^2^ ion at *m/z* 285 or 287 [M − H]^–^ and 287 or 289 [M + H]^+^, as major fragments, correspond to that of kaempferol or dihydrokaempferol, in positive and negative ion modes, respectively. Peak 11 with [M − H]^–^; *m/z* 461 was identified as kaempferol-7-O-glucuronide, after conjugation with a gluconyl at *m/z* 285 [M − H-gluconide]^−^ and RDA fragment ions of the aglycone. The other major fragment ion *m/z* 229 [M + H-H_2_O-2CO-OH]^+^ for 289 [M + H]^+^ of dihydrokaempferol at peaks 45 and 53, or on its methoxy kaempferol derivative at peaks 50 and 52. Peak 17 was identified as quercetin 3^1^-O-glucuronide after displaying [M − H]^–^; *m/z* 477 and MS^2^ fragments *m/z* 301 for the quercetin aglycone, after losing 176 Da of the glucuronide sugar. Flavanones give weak RDA fragments and commonly lose ring B by breaking at different positions [51]. They were identified at peaks 9, 10, 18, 19, 26, and 49. Peak 18 and 19 displayed [M − H]^–^; *m/z* 287 and the MS^2^ main fragments 151 [^1,3^A^–^], formed through the retrocyclization RDA cleavages of the C-ring of the aglycone involving 1 and 3 bonds (bonds 1 and 3 refer to the O—C-2 and C-3—C-4 bonds of the C-ring) [51], which is consistent with that of the aglycone eriodictyol. The conjugation with hexuronide (176 Da), formed an ion at [M − H]^–^; *m/z* 463 and thus was tentatively identified as eriodictyol-O-hexuronide for peaks 9 and 16. Identification of eriodictyol and eriodictyol-O-hexuronide in *E. africanus,* is consistent with an earlier report [28]. The compound in peak 26 was identified as naringenin, due to [M − H]^−^; *m/z* 271 and the MS^2^ characteristic fragment ions *m/z* 227 [M − H-CO_2_]^−^. Peak 49 showed [M + H]^+^; *m/z* 271 with the MS^2^ fragment ions 153 [^1,3^A^+^] (RDA cleavages of the C-ring of the aglycone) and 163 [^0,4^B^+^], (bonds 0 and 4 refer to the O—C-1 and C-4—C-3 bonds of the C-ring) which are characteristic of the flavanone fragmentation scheme [51] and thus identified as 3″,4″,7-trihydroxy flavanone.

#### 2.3.4. Analysis of the Coumarins and Furanocoumarins

Seventeen coumarins and furanocoumarins were detected at peaks 34, 35, 37, 38, 39, 40, 41, 42, 43, 44, 54, 57, 58, 59, 60, 61, and 62. Most of them exhibited a UV λ_max_ at 274 and 312 nm, characteristic of coumarin. Peaks 35 and 37 were identified as that of umbelliferone (hydroxy coumarin), consistent with the earlier literature [52]. Peak 59 was therefore identified as 6-(3,3-Dimethyl)-7-hydroxycoumarin, consistent with Yang et al. [52]. Peaks 34 and 44 were identified to be those of a furanocoumarin and xanthotoxin. This was because of the formation of [M + H]^+^; *m/z* 217 and that of the MS/MS fragment *m/z* 203 [xanthotoxol + H]^+^; consistent with the earlier literature [52]. Its derivative was formed at peak 57. Another furanocoumarin isopimpinellin was identified at peaks 38, 42, and 43 with the [M + H]^+^ ion shown at *m/z* 247 and the MS/MS fragments *m/z* 217, 229, 183, and 171. Peak 40 was identified as leptophyllin with [M + H]^+^; *m/z* 263 and the MS^2^ fragments *m/z* 217 [M + H-H_2_O-CO_2_]^+^, 91 [M + H-C_3_H_8_O-4CO]^+^, 203 [M + H-C_3_H_8_O]^+^. The existence of *m/z* 203, 217 and 91 suggested that the compound in peak 40 could have the skeleton of xanthotoxin [52]. Peaks 54, 58, 60, and 62 showed [M + H]^+^; *m/z* 213 and its fragmentation behavior is similar to that of 6-(3,3-Dimethylallyl)-7-hydroxycoumarin [52].

#### 2.3.5. UPLC-QTOF-MS Quantitation of the Phenolic Compounds

**Figure 4:**
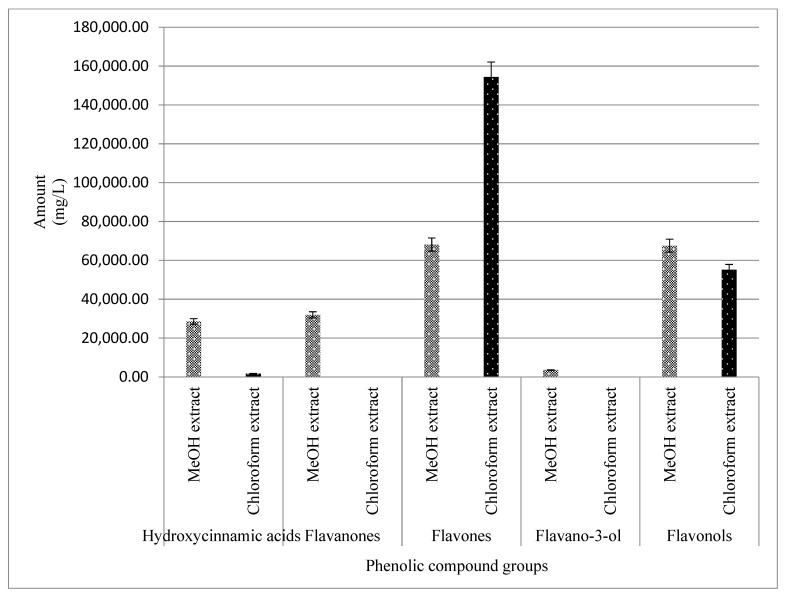
Amount of phenolic compounds quantitated from the methanol and chloroform extracts of *E. africanus*.

To improve on the impact of the study, the phenolic compounds were quantified using marker compounds, as described earlier [21]. The UV/vis absorptions, capable of distinguishing the phenolic subclasses, were considered a starting point for compound quantification. Based on the qualitative analysis, four chemical markers, namely phenolic acids and derivatives (neochlorogenic acid (peak 1), caffeic acid (peak 3), ferulic acid (peak 6), and coumaric acid (peak 5)), flavonol (peak 7), dihydrochalcone (peak 8), and flavano-3-ols (peak 2/4), were selected for the simultaneous quantitative determination (Figure 4). They were the most abundant in many reported plant methanol and chloroform extracts. They have been well represented in this study in the methanol and chloroform extracts of *E. africanus*. Based on the UV spectrum of the markers, the UV detection wavelengths were chosen. The phenolic acids and the derivatives had the strongest UV absorption at 300, 308, and 325 nm, whereas flavonols and flavanones at 254, 255, and 354 nm, flavones at 284 and 350 nm, and finally flavan-3-ol at 277 nm. The limits of quantification and detection, LOQ and LOD, respectively, presented in Table 4 were calculated by the parameters of the analytical curves (standard deviation of the response and slope) in our previous studies [21]. The standard deviation of the y-intercepts of the regression lines was used as the standard deviation of the blank. The LODs and LOQs were estimated as 3.3 and 10 times the standard deviation of the blank/slope ratio of the calibration curve, respectively. According to the LODs and LOQs, the phenolic compounds were highly detectable and quantifiable using the methods specified in the 70% ethanol extract of *E. africanus* (Figure 4). (Table 4). This approach may be used to profile the phenolic compounds from the samples examined, according to the observed limits of detection and quantification. Neochlorogenic acid, caffeic acid, ferulic acid, coumaric acid, rutin, catechin, epicatechin, and phloridzin equivalents have been used to express the quantity of the phenolic constituents like in the previous studies [21,53].

### 2.4. Alpha-Glucosidase Inhibition

**Figure 5:**
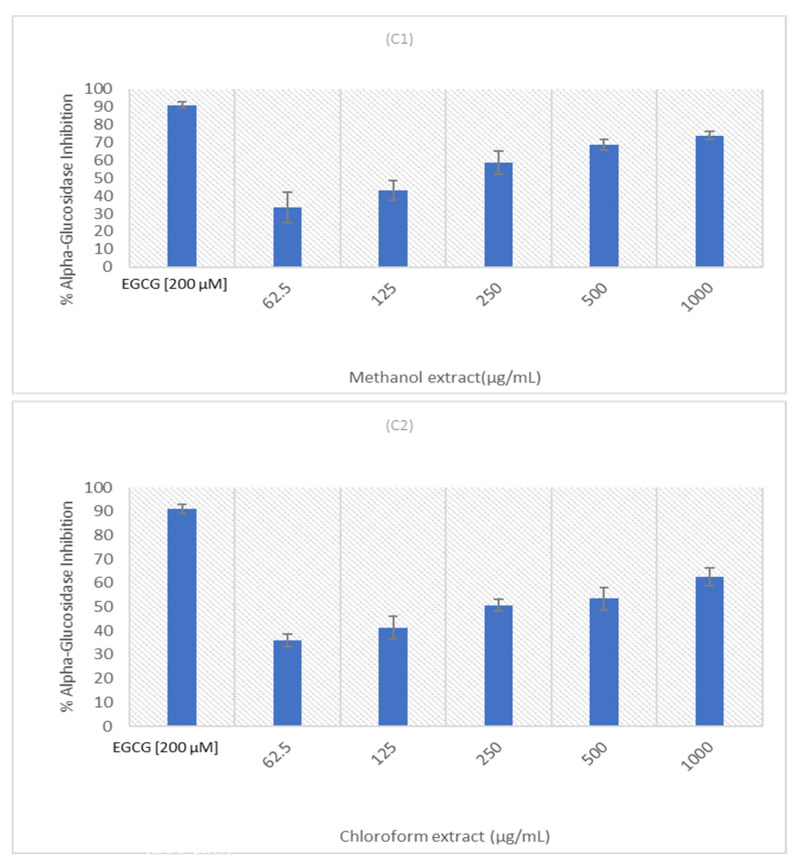
α-glucosidase inhibition of three samples. Epigallocatechin Gallate (ECGC) [200 µM] was used as a positive control. Error bars indicate the standard deviation of the mean of the three replicates from a single experiment.

Natural plant products are well-known for their multipurpose uses as preventative and therapeutic agents, as well as their low toxicity and adverse effects [54]. The adverse effects of diabetes drugs necessitated the development of innovative approaches for DM treatment [55]. Multiple studies have shown that plant extracts can function as α-glucosidase inhibitors, implying that they can be used to alleviate hyperglycemia [55,56,57]. The alpha-glucosidase inhibition of the crude methanolic and chloroform extracts of *E. africanus* was investigated and the results are depicted in Figure 5. It was observed that both extracts exhibited dose-dependent alpha-glucosidase inhibitory activities. Each of them displayed a similar inhibitory ability, with the sample methanolic extract exhibiting a higher inhibition of α-glucosidase (above 70%) at its highest tested concentration of 1000 μg/mL. The IC_50_ of these extracts are estimated as follows: the methanolic extract between 125 and 250 μg/mL, while the chloroform extract is approximately 250 μg/mL. A dose-dependent manner of the α-glucosidase inhibition has been reported by other researchers on some plant extracts in the literature [58]. Previous studies have shown that plant extracts that exhibited excellent antioxidant activities are more likely to demonstrate an α-glucosidase inhibition, depending on the type of active phytochemical compounds, which can vary from species to species, and rely on extraction procedures [59,60].

The results of this study are consistent with the findings of Bhatia et al. [54], who found a methanolic extract of *Cornus capitata* that displayed an inhibitory activity with a 98.37% inhibition (IC_50_ 12.5 μg/mL). In nature, the phenolics and flavonoids have the capacity to neutralize the free radicals (ROS) and can block the α-glucosidase to regulate blood sugar levels [61]. As previously documented, the antidiabetic effects of plant extracts were most likely related to the polyphenols found in plant extracts, and the inhibition of α-glucosidase was identified as the key target for the treatment of high postprandial blood glucose levels [62,63]. The final stage of starch digestion is catalyzed by α-glucosidase, which hydrolyzes the terminal glucose molecules from the non-reducing ends of oligosaccharides. α-glucosidase, also known as maltase-glucoamylase (MGAM), and sucrose-isomaltase (SI), which is a membrane-bound enzyme found near the brush border of the epithelial cells of the small intestine [64,65].

Alpha-glucosidase inhibitors slow the rate at which glucose may be absorbed and distributed in the body, by slowing down the digestion of carbohydrates and this prevents postprandial hyperglycaemia. For α-glucosidase inhibitors to successfully prevent the hydrolysis of oligosaccharides, they must ideally bind to all four catalytic domains of the enzyme. Acarbose is one such inhibitor that functions via a competitive inhibition mechanism [66]. As a result, α-glucosidase inhibitors are widely used as oral antidiabetic drugs in the early stages of T2D, to treat obesity and postprandial hyperglycemia. Several studies have reported that flavonoids have potential inhibitory effects on α-glucosidase [67,68,69]. Furthermore, it has been established that α-glucosidase inhibitors have a protective influence on the blood vessels by lowering the postprandial glucose levels, which are linked to endothelial dysfunction, cardiovascular disease, and stroke [70].

In the present study, the quercetin conjugates, eriodictyol, luteolin, apigenin-7-glucoside, apigenin, narigenin, vitexin, chlorogenic acid, and coumarins, such as leptophyllin and xanthotoxol, have been detected in the methanol extracts, which are reported to be strong α-glucosidase inhibitors [71,72,73,74,75]. Eriodictyol also suppresses the activation of the NF-κB system and reduces TNF-α, the intercellular adhesion molecule 1 (ICAM-1), vascular endothelial growth factor (VEGF), and endothelial NOS (eNOS) [76]. Quercetin showed IC_50_ = 0.53 mmol/L, vs. 1.7 mmol/L for acarbose, a positive control [72]. Vitexin, one of the compounds identified in this study, has previously demonstrated an inhibitory activity against α-glucosidase obtained from rat intestines with IC_50_ of 0.51 mmol/L [73]. Many polyphenols and coumarins with similar skeletons to the ones identified here have shown a promising α-glucosidase inhibitory activity. For example, coumarin compounds isolated from the root extract of *Rosa rugosa,* showed a potent sucrase inhibitory activity (61.88 ± 3.19% to 84.70 ± 3.07%) at a concentration of 1.0 mmol/L (IC_50_ ranging between 0.25 ± 0.04 to 0.48 ± 0.12 mmol/L), and the activity was comparable with that of acarbose (50.96 ± 2.97% inhibition, IC_50_ < 0.5 mmol/L) [77]. Gallic acid, an important constituent of many plant species [78], showed a strong inhibitory activity against glucosidase, both in vitro and in vivo; its IC_50_ value (24.3 mol/L) was lower than that of acarbose (59.5 mol/L). Methyl gallate obtained from the dried stem and bark extracts of *Terminalia superb* (IC_50_ = 11.5 mol/L), showed a strong α-glucosidase inhibitory activity [79]. Luteolin and luteolin- 7-glucoside have shown a high inhibitory activity against α-glucosidase and α-amylase [75,80]. In addition, apigenin-7-glucoside (IC_50_ = 0.17 ± 0.005 Luteolin (IC_50_ = 0.17 ± 0.026) and chlorogenic acid (IC_50_ = 1.4 ± 0.03) have previously demonstrated a considerable inhibitory activity, compared to acarbose, the positive control (IC_50_ = 0.023 ± 0.002) [74]. All these compounds have been identified in both the methanol and chloroform extracts of *E. africanus.*

### 2.5. Glucose Uptake

Medicinal plants are used in the treatment of diabetes, and this has generated attention, due to their efficiency and cost-effectiveness [56]. In the search for plant-based products for managing diabetes, the effect of crude methanolic and chloroform extracts of *E. africanus* was investigated on the glucose uptake and utilization in C3A hepatocytes and L6 myoblasts. Following the 24 h chronic exposure to the samples, the glucose uptake was determined over a 4 h period, as a function of the amount of glucose in the spent culture medium. The positive control, insulin, was added just prior to the experiment to represent an acute effect. The glucose utilization was measured after 24 h of treatment and reflects the cumulative change in the concentration of the glucose during treatment. Considering that glucose absorption through the glucose transporter proteins is the rate-limiting step for the cellular glucose uptake, it may be assumed that the amount of glucose left over indirectly reflects the glucose uptake, even when the intracellular glucose concentration is not known.

**Figure 6:**
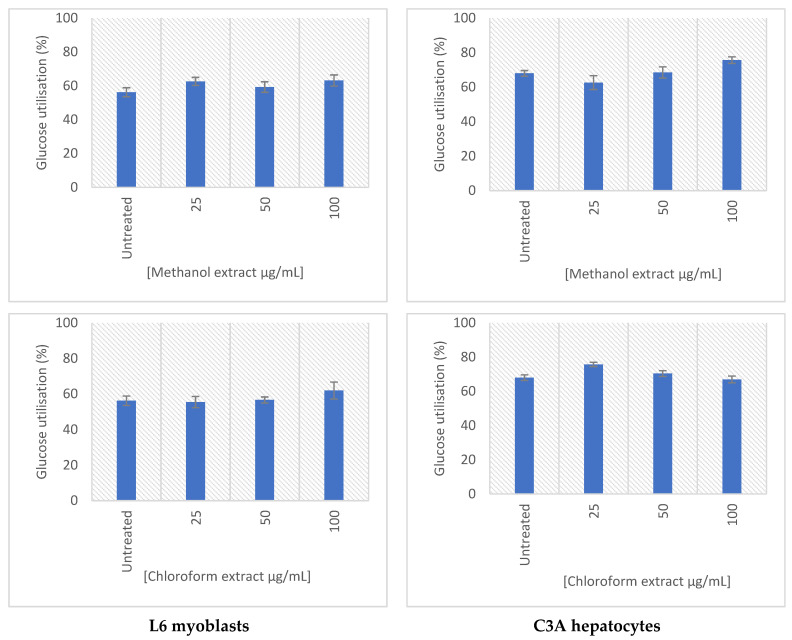
Glucose utilization (%) after 24 h of treatment in the L6 myoblasts (**left**) and the C3A hepatocytes (**right**). Results were normalized to the cell viability, as determined using the MTT assay. Error bars indicate the standard deviation of the mean of four replicates from a single experiment.

**Figure 7:**
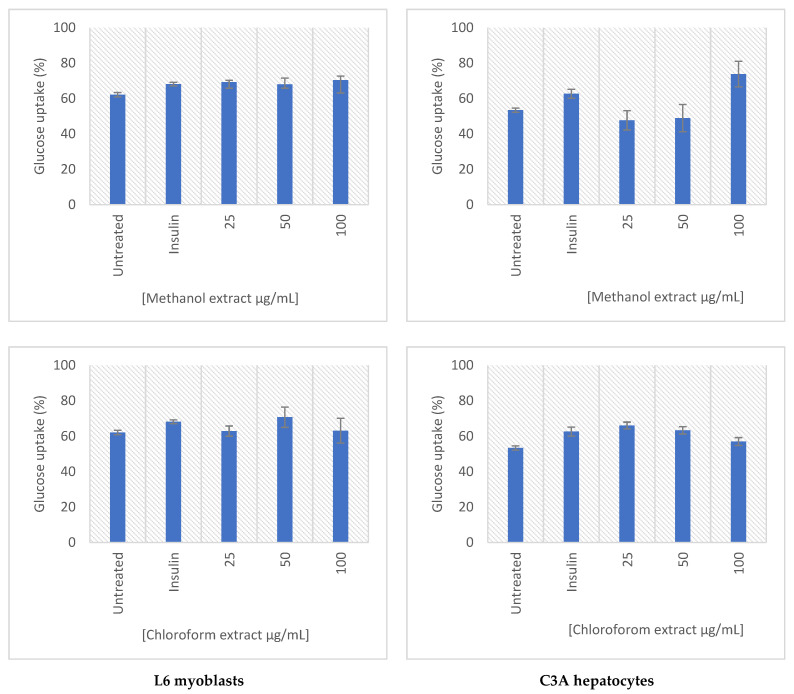
Glucose uptake (%) after 4 h in the L6 myoblasts (**left**) and C3A hepatocytes (**right**), following a 24-h treatment period. Results were normalized to the cell viability as determined using the MTT assay. Error bars indicate the standard deviation of the mean of four replicates from a single experiment.

The effect of the extracts on the glucose utilization and the uptake was determined in C3A and L6 cells (Figure 6). Both extracts demonstrated a dose-dependent increase in glucose uptake in both cell lines tested. The methanolic extract of *E. africanus* exhibited an increased glucose utilization in L6 myoblasts at all tested concentrations (25–100 μg/mL), whereas chloroform extract of *E. africanus* showed no significant effects. In the C3A hepatocytes, the methanolic extract showed an increase in the glucose utilization at a treatment concentration of 100 μg/mL, while the chloroform extract was at 25–50 μg/mL. Moreover, the methanolic extract increased the glucose uptake in the L6 myoblasts at all tested concentrations (25–100 μg/mL), while the chloroform extract displaced the glucose uptake from 50–100 μg/mL. No significant cytotoxic effects were observed for all test samples in the L6 cells or C3A cells (Figure 7). The main site for utilizing the glucose following meals is skeletal muscle, which also plays a significant role in maintaining the glucose homeostasis [81]. Numerous compounds produced by plants have been attributed to the positive impact on the glucose transport and metabolism in the skeletal muscle cells. This study demonstrated that treatment with a plant extract boosted the glucose absorption in a concentration-dependent manner, indicating that the higher the drug concentration, the stronger the effect [82]. Considering that the adipocytes are the primary site of the insulin action, they are crucial for both the control of the whole-body glucose homeostasis and glucose metabolism [83]. It is important to mention that the biological effect exhibited by these extracts could be due to the synergistic effect of the bioactive compounds identified in them.

### 2.6. Pancreatic β-Cell Proliferation

**Figure 8:**
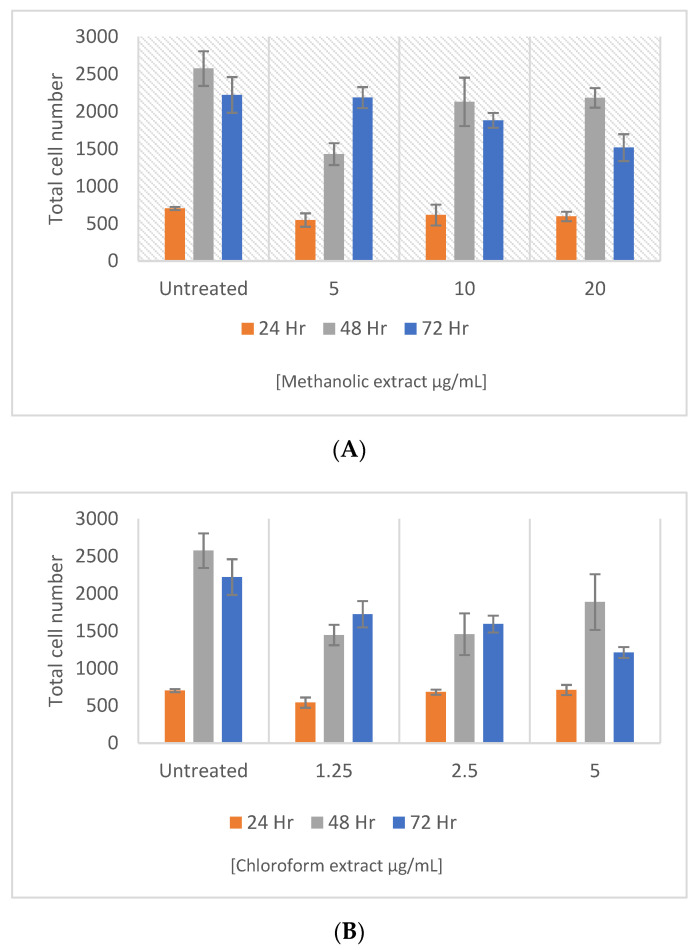
Effect of three samples on the INS-1 pancreatic β-cell proliferation. Cells were stained with Hoechst 33342 and the total cell number was determined after 24, 48, and 72 h of treatment with samples of the methanol extract (**A**) and chloroform extract (**B**). Error bars indicate the standard deviation of the mean of four replicates from a single experiment.

The beta cell apoptosis is a hallmark of both type 1 and types 2 diabetes [84,85]. The islets of the pancreas’ beta cells undergo a selective apoptosis, which results in an insulin insufficiency and persistent hyperglycaemia in type 1 diabetes (T1DM). In type 2 diabetes (T2DM), the functional deficiencies and reduced beta cell mass both lead to a beta cell failure [86]. Once T2DM is present, abnormal amounts of metabolic factors cause the beta cell death, which is followed by apoptosis [87]. Strong activators of the β-cell proliferation can restore the pancreatic function through neogenesis [88]. Therefore, showing the therapeutic potential in the relief and or reversal of both the T1DM and T2DM symptoms. As a result, in the present study, the potential of methanolic and chloroform extracts of *E. africanus* were investigated (Figure 8). Unfortunately, none of the extracts exhibited a significant ability to stimulate the β-cell proliferation, instead a decrease in the total cell number was observed, relative to the untreated control after 72 h. This implies that the hypoglycaemia potential of both the methanolic and chloroform extracts of *E. africanus* is not achieved through the activation of the β-cell proliferation, unlike those reported in the literature by some researchers [89,90,91,92,93].

Some phenolic compounds, such as kaempferols and dihydrokaempferol, and some of their conjugates, such as kaempferol glucosides, quercetin, and catechin affect the glucose homeostasis. Kaempferol glycosides improves the glucose homeostasis by enhancing the pancreatic β-Cell function [94]. The phenolic compounds, such as quercetin and catechin amplified the proliferation inhibition of the pancreatic β-cell [94]. The pancreatic β-cell exhibit a sensitivity to oxidative stress, a factor that may lead to the impairment of the β-cell functioning, and thus diabetes.

### 2.7. In Vitro Anti-Inflammatory Activity

Although inflammation is typically thought of as a protective or healing response, many chronic illnesses are marked by persistent inflammation, that leads to tissue dysfunction [95,96,97]. For this rationale, to effectively assess the potential therapeutic importance, the anti- or pro-inflammatory activity of the test samples must be considered within the context of the disease in question, as well as the disease development stage at which intervention would be considered. The mouse macrophage cell line RAW 264.7, which has been extensively studied and is a common model for examining the anti-inflammatory properties of the test extracts, was employed in the present study.

**Figure 9:**
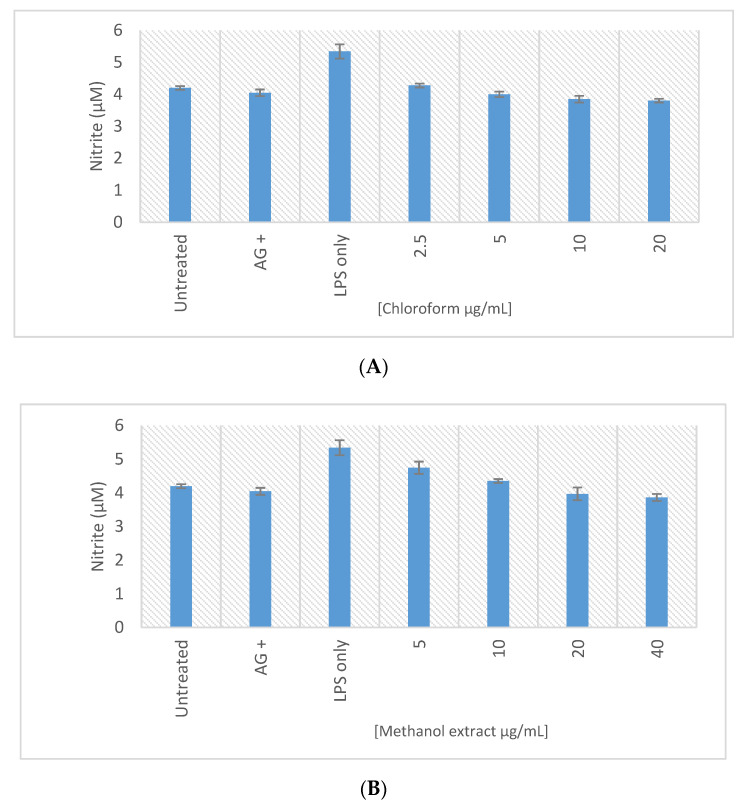
Nitric oxide production in the LPS activated macrophages treated with samples of the methanol extract (**A**) and chloroform extract (**B**). Bar graph represents the quadruplicate values of one experiment. Error bars represent the standard deviation of the mean.

**Figure 10:**
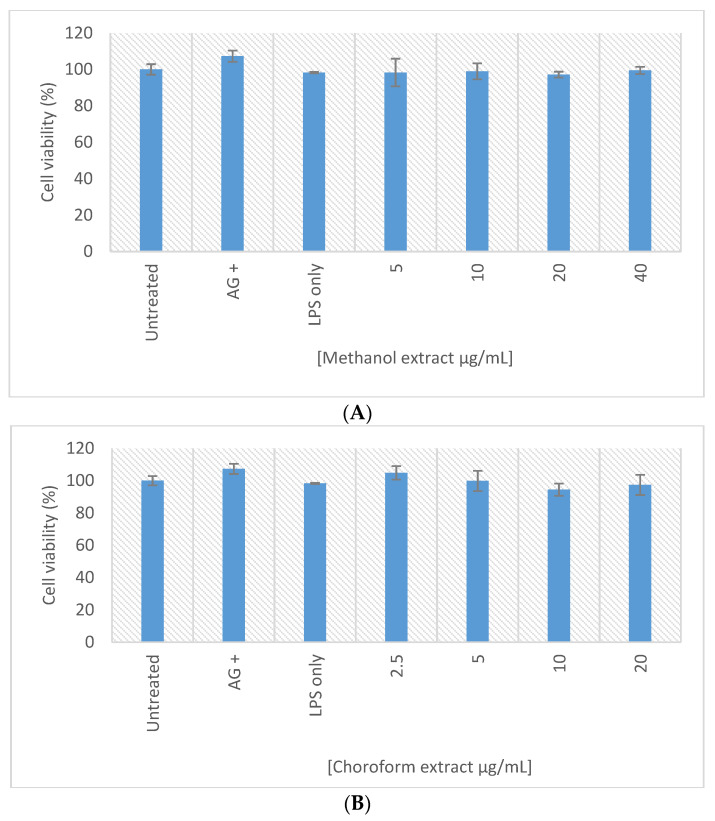
Cell viability (%) of the LPS activated macrophages after 24 h exposure to samples of the methanol extract (**A**) and chloroform extract (**B**). Bar graph represents the quadruplicate values of one experiment. Error bars represent the standard deviation of the mean.

The anti-inflammatory activity of the methanolic and chloroform extracts of *E. africanus* was assessed using the RAW 264.7 macrophages and the Griess assay. The cytotoxic effect of the extracts on the RAWs was also determined to accurately establish the potential anti-inflammatory activity. The anti-inflammatory activity is indicated by the decrease in the nitrite concentration, in response to LPS activation of the RAW macrophages, with no effect on the cell viability. The methanolic extract of *E. africanus* exhibited the anti-inflammatory activity from a concentration of 10 µg/mL, while the chloroform extract of *E. africanus* from 2.5 µg/mL (Figure 9), and interestingly, no significant cytotoxicity was observed from both extracts (Figure 10). The results from the present study concur with the findings of other researchers, reported in the literature [98]. The inflammatory cytokines, such as IL-1, which cause the beta cell toxicity, are produced, and released because of hyperglycaemia [99]. When the process of glucose intolerance results in overeating, the lipids stored in the non-adipose tissue are ineffectively oxidized; as a result, their products and ceramide promote the nitric oxide generation and induce death in the pancreatic cells [100]. 4,5-Di-O-Caffeoylquinic acid suppresses the inflammatory responses through the TRPV1 activation and was detected in the methanol extract [75].

### 2.8. In Vitro Macrophage Activation Screening

**Figure 11:**
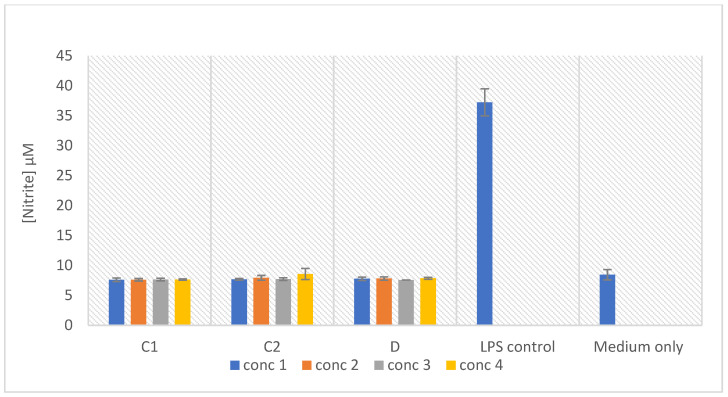
Nitric oxide production in the macrophages treated with different concentrations of the samples, as indicated in Section 2.2. Concentrations tested were as follows: Sample C1 (methanol extract): 5, 10, 20, and 40 µg/mL; Sample C2 (chloroform extract): 1.25, 2.5, 5, and 10 µg/mL. Bar graph represents the quadruplicate values of one experiment. Error bars represent the standard deviation of the mean.

**Figure 12:**
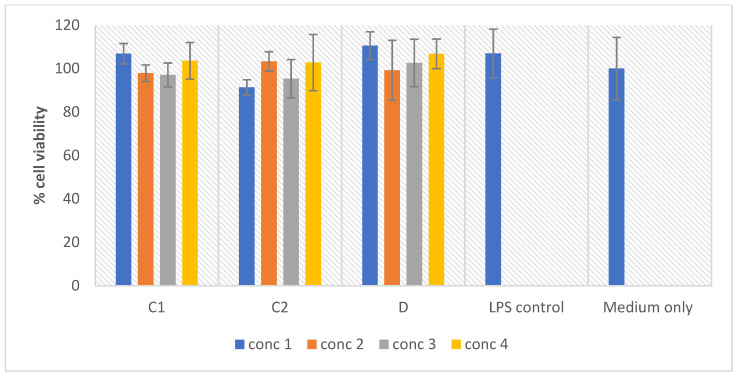
Cell viability (%) of the LPS activated macrophages after 24 h exposure to the treatments. Concentrations tested were as follows: Sample C1 (methanol extract): 5, 10, 20, and 40 µg/mL; Sample C2 (chloroform extract): 1.25, 2.5, 5, and 10 µg/mL. Bar graph represents the quadruplicate values of one experiment. Error bars represent the standard deviation of the mean.

The outstanding prospects for preventative and therapeutical approaches with minimal adverse side effects are the natural compounds derived from medicinal plants [100,101,102]. A well-established model used for analyzing the anti-inflammatory and macrophage activation capabilities of the test samples is the murine macrophage cell line RAW 264.7. The plant extracts were screened against the RAW 264.7 cells for the macrophage activation potential and the results are represented in Figure 11. The macrophage activation is indicated by an increase in the nitrite concentration, in response to the treatment of the RAW macrophages with no effect on the cell viability, as seen with the LPS treated cells. The plant extracts do not display the potential to activate the macrophages, as compared to the control. However, no toxic effect was recorded at the tested concentrations (Figure 12). These results revealed that the anti-inflammatory activity demonstrated by the extracts described above, was not achieved through the activation of the macrophages.

## 3. Materials and Methods

### 3.1. Plant Collection and Identification

The fresh leaves of *E. africanus* were collected behind the Department of Horticultural Science and Textiles building (Coordinate: −33.87507, 18.637135) at Cape Peninsula University of Technology, Bellville campus, South Africa. The plant was authenticated by Prof. Learnmore Kambizi (a botanist at the Department of Horticultural Sciences) and a specimen with the voucher no: 3903 was deposited in their herbarium.

### 3.2. Plant Extraction

The leaves were thoroughly washed with tap and distilled water, to remove impurities, they were air-dried at room temperature for 10 days, and ground with a grinder. One hundred grams (100 g) of the leaf powder was extracted with 1500 mL of 70% methanol and chloroform, in two separate flasks, for 72 h, under constant stirring. The mixture was then filtered using a Whatman no. 1 filter paper and the supernatant was then transferred to a rotary evaporator, where the solvent was evaporated at 48 °C under reduced pressure, to collect the crude extract, which was kept at 4 °C, until further use [21].

### 3.3. Determination of the Total Polyphenol and Flavonol Contents

The Folin–Ciocalteu procedure described by Okafor et al. [103], was explored to determine the total polyphenol content of the extract. The total phenolic content of the extract was measured and represented as mg of the sample gallic acid equivalent (GAE)/g. The total flavonol content was determined using the method reported by Yermakov et al. [104]. The result was represented as milligram catechin equivalents (CE) per gram sample (g).

### 3.4. Estimation of the Antioxidant Capacity

The Trolox equivalent antioxidant capacity (TEAC) of the extract was evaluated using the method outlined by Re et al. [105]. Trolox was employed as a standard drug, and the results were represented as the micromole Trolox equivalent per gram sample (TE)/g. The Benzie and Strain [106] protocol was used for the FRAP analysis. Ascorbic acid was used as the standard antioxidant drug. The potential of the plant extract to scavenge the DPPH radicals was tested using the 2,2-diphenyl-1-picrylhydrazyl (DPPH) assay established by Ngxabi et al. [107]. The results were expressed as M/Trolox equivalent per gram dry weight (mol TE/g). The results were reported as M/Trolox equivalent per gram dry weight (mol TE/g).

### 3.5. Alpha-Glucosidase Inhibition

The alpha-glucosidase inhibition assay was conducted, as described by van de Venter et al. [108]. Briefly, “the plant extracts were sonicated and diluted in an assay buffer (67 mM potassium monobasic anhydrous phosphate pH 6.8) to concentrations of 1000, 500, 250, 125, and 62.5 µg/mL from a final concentration of 100 mg/mL of the dimethyl sulfoxide (DMSO) plant extracts. Thereafter, the extracts were performed in a stepwise manner in 96-well plates, in triplicate. Ten microliters of each extract were added to each well, followed by 70 microliters of the enzyme (*Saccharomyces cerevisiae*’s α-glucosidase), which was then incubated at 37 °C for 10 min. Twenty microliters of the substrate (10 mM p-Nitrophenyl-D-glucopyranoside) was then added, and the mixture was incubated at 37 °C for 20 min and the reaction was terminated by adding 25 μL of Na_2_CO_3_ (100 mM). The assay was carried out alongside with the positive control drug, epigallocatechin gallate (EGCG)”. Using a BioTek^®^ PowerWave XS spectrophotometer (Winooski, VT, USA), the absorbance of the mixture was read at 410 nm. The percentage of the α-glucosidase inhibition was estimated as follows in the absence of enzyme and substrate controls:

% α-glucosidase *inhibition* = ((A410 nm of control − A410 nm of test sample))/(A410nm of control) × 100

### 3.6. Glucose Uptake

#### 3.6.1. Cell Line Maintenance

From the Japanese Collection of Research Bioresources Cell Bank, L6 rat myoblast cells were acquired (Osaka, Japan). The routine maintenance of the cells involved maintaining them in 10 cm culture dishes with full media (high glucose DMEM, with 10% FBS and 1% Pen-Strep) and incubating them at 37 °C in a humid environment with 5% CO_2_. The American Type Culture Collection provided human hepatoma-derived C3A hepatocytes (ATCC, Manassas, VA, USA). A complete medium (MEM with 1% NEAA, 10% FBS, and 1% Pen-Strep) was used to maintain the cells in 10 cm culture dishes. The cells were kept at 37 °C in a humidified environment with 5% CO_2_ during maintenance.

#### 3.6.2. Glucose Utilization (A) and Uptake (B)

The glucose uptake experiment was carried out with a few modifications, as described by van de Venter et al. [108]. Briefly, “in dimethyl sulfoxide (DMSO), the extracts were reconstituted at a stock concentration of 100 mg/mL. The extracts were sonicated and kept in a freezer until needed. The cells were seeded in 96 well plates (2 × 10^4^ cells/well, 100 μL aliquots) and left overnight to attach. Thereafter, the treatments were prepared in a complete medium, added to the cells, and incubated for 24 h. Five microliters (5 μL) of used culture/treatment media were removed and transferred to clean 96-well plates, sealed, and kept at −20 °C until needed (A). The cells were washed with 100 μL of PBS after the leftover media was aspirated. Insulin (1 μg/mL) was employed as a positive control, and 25 μL of the incubation buffer (RPMI-1640 mixed with PBS containing 0.1% BSA to a final glucose concentration of 8 mM) was added to the cells. Following 4 h of incubation, the cells were transferred with 5 μL of the culture media to a fresh 96-well plate (B).

A colorimetric glucose oxidase/peroxidase assay, based on the method described by Trinder [109], was used to determine the changes in the concentration of glucose in the spent culture medium, as a function of the red-colored quinoamine dye complex produced, determined spectrophotometrically at 510 nm. Briefly, “the glucose oxidase assay reagent was freshly prepared, as follows: 3 mM phenol, 0.4 mM 4-aminoantipyrine, 0.25 mM EDTA and 2.5 U/mL horseradish peroxidase in 0.5 M PBS (pH 7.0) with 1 mU/mL glucose oxidase from *Aspergillus niger*. Two hundred microliters (200 μL) of a glucose oxidase reagent was added to the plates (A and B). The reaction was incubated for 15 min at room temperature. The absorbance was measured at 510 nm using a BioTek^®^ PowerWave XS spectrophotometer (Winooski, VT, USA). The cell-free wells containing the incubation buffer and complete culture mediums were used as the glucose standards. The glucose uptake and utilization were determined as a function of the concentration of the glucose (mM) remaining and expressed as the difference between the mean of the standard and test extracts.

### 3.7. 3-4,5-dimethylthiazol-2,5-diphenyltetrazolium Bromide (MTT) Assay

A MTT assay was performed to ensure that any observed changes in the glucose utilization or uptake were not due to changes in the cell viability. The MTT assay is based on the reduction of a yellow water-soluble tetrazolium salt to an insoluble purple formazan product. The purple-colored formazan crystals are dissolved in dimethyl sulfoxide (DMSO), and the absorbance was measured at 540 nm. The concentration of formazan is then related to the number of healthy viable cells [110,111]. Briefly, the remaining treatment medium from the assay described above was aspirated from all wells and 100 μL of the complete medium, containing 0.5 mg/mL MTT, was added. Thereafter, the cells were incubated for 1 h at 37 °C and a MTT medium was removed and 100 μL DMSO was added to each well. The absorbance was read at 540 nm using a BioTek^®^ PowerWave XS spectrophotometer (Winooski, VT, USA).

### 3.8. Pancreatic β-Cell Proliferation

#### 3.8.1. Cell Line Maintenance

The INS-1 pancreatic beta-cells from rat insulinomas were provided by Prof. Rutter from the University of Bristol in England. RPMI-1640 with 10% FBS was used to maintain the cells in 10 cm culture dishes, and they were kept at 37 °C in a humid environment with 5% CO_2_ for the whole incubation period.

#### 3.8.2. β-Cell Proliferation Assay

The β-cell proliferation assay was performed, as described by Pringle et al. [88]. Briefly, the cells were seeded in 96-well plates at a density of 2 × 10^4^ cells/well in 100 μL aliquots and left overnight to attach. The treatments were prepared in complete medium (RPMI-1640 with 10% FBS) to concentrations of 20, 10, and 5 μg/mL for the methanol extract and 5, 2.5, and 1.25 μg/mL for the chloroform extract, and subsequently, the cells were incubated for 24, 48, and 72 h. Following each incubation period, the culture/treatment medium was gently aspirated, and a 50 μL staining solution [Bis-benzamide H 33342 trihydrochloride (Hoechst) (5 μg/mL) in PBS with Ca^2+^ and Mg^2+^] was added to each well. Subsequently, the plates were incubated at 37 °C for 30 min. Following the incubation period, the fluorescent micrographs were captured with an ImageXpress Micro XLS Widefield Microscope (Molecular Devices) with a 10× Plan Fluor objective and DAPI filter cube. The total number of cells was calculated by analyzing the acquired pictures with the MetaXpress program and the multi-wavelength cell scoring application module.

### 3.9. In Vitro Macrophage Activation

Briefly, the in vitro macrophage activation assay was conducted, following the description of Rampa et al. [112]. Briefly, dimethyl sulfoxide (DMSO) was used to reconstitute the plant extracts to a stock concentration of 100 mg/mL., and the RAW 264.7 cells (Cellonex, South Africa) were seeded at a density of 1 × 10^5^ cells per well in 96-well plates containing the RPMI1640 culture media supplemented with 10% FBS (RPMI complete medium), and left overnight to attach. The spent culture media from the previous day was removed, and 50 µL sample aliquots were added and diluted in the RPMI complete medium, to achieve the final concentrations (methanolic extract: 5, 10, 20, and 40 µg/mL; chloroform extract: 1.25, 2.5, 5, and 10 µg/mL). Lipopolysaccharide (LPS) serves as a positive control drug at 500 ng/mL. Subsequently, the cells were incubated for 24 h. The manufacturer’s instructions were followed to make the sulfanilamide solution and the NED solution, and 50 μL of the used culture medium was transferred to a fresh 96-well plate to measure the NO production. The used culture media was then mixed with 50 μL of the sulfanilamide solution and allowed to incubate for 10 min in the dark at room temperature. Thereafter, 50 microliters (50 μL) of the NED solution were added to each well, and each well was then incubated for a further 5–10 min, at room temperature and in the dark. Using a BioTek^®^ PowerWave XS spectrophotometer, the absorbance was read at 540 nm. The concentration of the NO in each sample was estimated using a standard curve constructed using sodium nitrite dissolved in a culture medium. Furthermore, to ascertain that toxicity was not a significant factor, the MTT assay was explored to test the cell viability, as described by van de Venter et al. [108] with minor modifications.

### 3.10. UPLC-ESI-QTOF-MS Analysis of the Methanol and Chloroform Extracts of E. africanus

The LC-MS analysis was conducted using a QA Waters Synapt G2 quadrupole time-of-flight mass spectrometer. It was fitted with a Waters ultra-pressure liquid chromatography (UPLC-MS) using Waters msE technology and photodiode array detection. The phenolic method and specification of the instrument were reported by Stander et al. [113], in the negative ion mode with minor modifications. The solvents A and B in the positive ion mode each contained 0.1% formic acid, while the mobile phase in this mode was made up of water and acetonitrile. Following 0.5 min of 100% solvent A, the gradient switched to 100% B for over 0.5 min to 12.5 min. Thereafter, 13 min into the runtime, it then changed to 100% A for the following 2 min in a total run time of 15 min. The flow rate was 0.4 mL/min, the seal wash was 5 min, and the column temperature was maintained at 55 °C. The ionizing electrospray 275 °C desolvation temperature, the 15 V cone voltage, and ESI Pos. Leucine encephalin was injected as a lock mass in the background, and sodium formate was employed for the calibration to obtain the precise mass measurements. The MassLynx software platform supplied with Waters mass spectrometers was used for manually processing each chromatogram.

### 3.11. Identification of the Compounds Using UPLC-ESI-QTOF-MS

The identified metabolites were given preliminary names, based on the accurate mass matches that were automatically searched in the databases, such as Metlin, massBank, NIST, and other libraries, such as PubChem, mass fragmentation patterns of compounds searched in the databases and a number of carbon atoms for the isotope relative abundance. All compounds were identified as unidentified using the accurate mass match, if their accurate mass error (AME) was more than 5 ppm [114]. To identify a particular compound, based on the retention time, the mass fragmentation, and the ionization modes, a few standards of the phenolic compounds were spiked under identical LC/MS conditions (positive and negative ion modes). Considering that it was not possible to obtain all standards and many compounds could be detected using UPLC-MS, the MS and MS2 fragment ions of other compounds, that were similar to those being annotated, were employed. The compound structures were elucidated using the MS-MS analysis of the sample’s compounds that were fragmented to match the product ion mass spectra. If isotope abundances were available, the number of carbon atoms in the peak was computed as a final step. The false annotations were minimized by using the predicted number of carbon atoms in the putatively recognized molecule. Different concentrations of the standard mixture of the chemical markers (3.9, 7.8, 15.6, 31.3, 62.5, 125.0, and 250.0 mg/L), were injected for quantification. The linearity of the calibration curve was checked by plotting the peak areas against the series of standard solution concentrations (mg/L) and determining the correlation coefficient using a linear regression model. In all cases, the system was linear when r > 0.99.

## 4. Conclusions

In the present study, the phenolic compounds of *E. africanus* were profiled, and the antidiabetic and anti-inflammatory potentials of the methanolic extract and chloroform leave extracts were investigated in vitro. Both extracts demonstrated a dose-dependent manner for the alpha-glucosidase inhibition and the extracts also demonstrated the potential to increase the glucose utilization at different concentrations in both the L6 cells and C3A cells, and there were no significant cytotoxic effects. However, none of the extracts exhibited a significant ability to stimulate the β-cell proliferation. Moreover, both extracts displayed a strong anti-inflammatory activity with no significant cytotoxic effect, indicating the relative safety in the therapeutic use for humans. The presence of several phytochemical compounds identified in the extracts could be responsible for the biological activities demonstrated in the present study. It is noteworthy that the biological effects displayed by these extracts may perhaps be prompted by the synergistic interaction of the bioactive compounds identified in them. Therefore, further research on the isolation of the pure compounds from these extracts and conducting in vivo tests is encouraged. Overall, the results of this study indicate that *E. africanus* may be a source of new bioactive compounds to control hyperglycaemia in the treatment of type 2 diabetes mellitus. Thus, further studies, such as the in silico molecular docking of the identified compounds with carbohydrates’ digestive enzymes, as well as in vivo studies, are encouraged. Additionally, the targeted isolation of the bioactive compounds responsible for these biological activities is of much significance in studying the biological activities of this plant. Thus, the present findings would be useful for future research directions on the application of traditional medicinal plants in the development of nutraceuticals and pharmaceuticals.

## Figures and Tables

**Table 1:** Phenolic compounds and antioxidant capacity of the methanolic extract and chloroform crude extracts of *E. africanus*.

Extracts	Polyphenols (mg GAE/g)	Flavonols (mg QE/g)	FRAP (µmol AAE/g)	DPPH (µmol TE/g)	TEAC (µmol TE/g)
Methanolic extract	112 ± 2.81	76.12 ± 7.95	752.64 ± 89.0	812.18 ± 51.12	631.63 ± 17.42
Chloroform extract	39.93 ± 1.36	44.81 ± 3.74	107.10 ± 2.41	58.70 ± 5.18	118.63 ± 3.74

Trolox equivalent antioxidant capacity (TEAC), ferric reducing antioxidant power (FRAP).

**Table 2:** Phytochemicals screened from the methanol (C1) and chloroform (C2) extracts of *E. africanus* in the negative ion mode.

No	t_R_ (min)	UV λmax (nm)	*m*/*z* [M − H]^−^	MS/MS	Tentative Name		Identification
1	1.13		191.0549	119	p-Coumaric acid ethyl ester	C1	New
2	4.14	295	554.2621 ^Cl−^	355, 401, 113	Caffeoyl-hexuronide derivative	C1	[27]
3	7.11	243, 326	353.0859	191, 179	3-Caffeoylquinic acid (Neochlorogenic acid)	C1	Standard
4	9.10	242, 326 sh	353.0879	191	1-Caffeoylquinic acid/ Chlorogenic acid	C1	[27]
5	10.45	249, 318	305.0705	198	Epigallocatechin	C1	New
6	11.57	237, 270, 330	593.1514	171, 479, 353	Apigenin-6,8-di-C-glycoside	C1	New
7	12.05		249.1122	205, 231, 187	Gemfibrozil	C2	New
8	12.14	245, 330	367.1002	191, 173, 134	Feruloyl quinic acid	C1	New
9	14.60	281, 284	463.0887	287, 151, 135, 113	Eriodictyol-O-hexuronide	C1, C2	[27]
10	14.66	284 sh	463.0890	287	Eriodictyol-O-hexuronide	C1	[27]
11	15.00	255, 347	461.0755	285, 217, 175	Kaempferol-7-O-glucuronide	C1	New
12	16.11	245, 327 sh	515.1200	173 > 179, 191, 353	1,5-Dicaffeoylquinic acid	C1, C2	New
13	16.50	243, 320	515.1211	353	3,4-Dicaffeoylquinic acid	C2	[27]
14	16.82		447.0944	187	6-Hydroxyluteolin 7-O-rhamnoside	C2	New
15	17.21	243, 326	515.1209	353	3,5-Dicaffeoylquinic acid	C1	[27]
16	17.42	245, 323 sh	515.1176	173 > 179, 135, 191, 353	1,4-Dicaffeoylquinic acid	C1	[27]
17	18.20	288, 325	477.1053	301, 171, 145	Quercetin 3^1^-O-glucuronide	C1	New
18	19.40	288	287.0539	151, 135	Eriodictyol	C1	[27]
19	19.48	234, 289	287.0551	151, 135	Eriodictyol	C1	[27]
20	19.49	288	233.1181	189, 215, 221, 159, 203, 177	unknown	C2	New
21	20.20	251, 347	523.2188	285	Luteolin derivative	C1	New
22	20.30	255, 338	285.0404	187, 195	Luteolin	C1	[45]
23	20.76	250, 347	315.0484	300	Isorhamnetin	C1	New
24	20.82	252, 273, 346	315.0503	161, 300	Isorhamnetin	C2	New
25	21.58	236, 288	317.0667	289, 299, 195	Myricetin	C2	New
26	22.40	289	271.0585	227	Narigenin	C1	New
27	22.86	265, 337	269.0450	161, 151, 117	Apigenin	C1	[45]
28	23.40	237, 272, 333	299.0551	284	Chrysoeriol (3′-O-methylluteolin)	C1, C2	New
29	23.71	242, 275, 340	329.0661	315	Tricin	C1	New
30	24.07	247, 273, 339	343.0826	315, 299, 297, 227	3′-O-methyl tricin/3″,4″,5,7-tetramethoxyluteolin (methlut)	C1, C2	New
31	24.22	235, 275, 329	313.0720	253	3^1^,4^1^-dimethyluteolin	C2	New
32	24.40	255	445.2216	325, 339, 265, 311, 287, 150, 221	Apigenin-7-O-glucuronide	C1	New
33	24.51	252	431.2066	265, 150	Apigenin-6-C-hexose	C1, C2	New

^Cl−^ = [M + Cl]^−^ “New” means it has been reported from *E. africanus* for the first time, although it has been previously identified from other plant sources, as referenced in the main text. Provided reference means earlier reported from *E. africanus* species.

**Table 3:** Phytochemicals screened from the methanol (C1) and chloroform (C2) extracts of *E. africanus* in the positive ion mode.

No	t_R_ (min)	UV λmax (nm)	*m/z* [M + H]^+^	MS/MS	Assignment		Identification
34	0.71		217.069	203	Xanthotoxin	C1	New
35	2.70		163.0404	135, 145	Hydroxy coumarin		New
36	2.88	236, 326	188.0727	146	Trans-3-indoleacrylic acid	C1	New
37	3.01	242, 286, 304, 340	163.040	145, 135	Hydroxy coumarin	C1	New
38	3.33	271, 331	247.133	229, 183, 201	Isopimpinellin	C2	New
39	3.45	321 sh	380.208	163, 364	Xanthotoxin-Hydroxy coumarin adduct	C1	New
40	3.72	291, 323	263.130	217, 187, 203, 91, 245	Leptophyllin	C2	New
41	4.40	286, 327	422.219	211, 163	Unknown dimer	C1	New
42	4.62	312	247.134	217, 229, 183, 171	Isopimpinellin	C2	New
43	4.72	291, 321	247.135	217, 229	Isopimpinellin	C2	New
44	4.84	312, 323	217.124	171, 143	Xanthotoxin	C2	New
45	5.00	287	289.0723	229, 163	Dihydrokaempferol	C1	New
46	5.05	250, 293, 346	287.0566	229	Kaempferol	C1	New
47	5.12	250, 293, 346	317.068	302, 163	Isorhamnetin	C1	New
48	5.18	273, 346	406.223	317	Isorhamnetin-4″-O-malonyl	C1	New
49	5.60	268, 291, 331	271.0615	153, 163	3″,4″,7-trihydroxy flavanone		New
50	5.64	274, 330	301.0721	247, 229	Methoxy kaempferol	C1, C2	New
51	5.78	273, 346	331.08185	316, 301, 168	Tricin	C1, C2	New
52	5.90	271, 347	324.181 ^Na^	229, 247, 307, 183, 289, 247	Methoxy kaempferol	C1, C2	New
53	6.20	235 289, 329	289.1440	229, 249, 185	Dihydrokaempferol	C1, C2	New
54	6.27	287, 325	231.1384	185, 157	6-(3,3-Dimethylallyl)-7-hydroxycoumarin	C1, C2	New
55	6.50	274, 346	345.0994	229, 247	3″,4″,5,7-tetramethoxyluteolin (methlut)	C1, C2	New
56	7.02	275, 334	315.087	251	3^1^,4^1^-dimethyluteolin	C1, C2	New
57	7.20	244, 276, 335	359.1140	217, 235	Xanthotoxin derivative	C1, C2	New
58	7.76	241, 331	231.1385	185, 157, 213	6-(3,3-Dimethylallyl)-7-hydroxycoumarin	C1, C2	New
59	8.43	244, 329, 331	233.1543	187, 215	6-(3,3-Dimethyl)-7-hydroxycoumarin	C1, C2	New
60	9.06	247, 331	231.1385	185, 157	6-(3,3-Dimethylallyl)-7-hydroxycoumarin	C1	New
61	9.07	312, 335	331.1905	231, 175, 185, 213	6-(3,3-Dimethylallyl)-7-hydroxycoumarinderivative	C1, C2	New
62	9.55	242, 274	149.0248 231.1385	93	6-(3,3-Dimethylallyl)-7-hydroxycoumarin	C1, C2	New

* ^H^_2_^O^ = [M + H-H_2_O]^+^, ^Na^ = [M + Na]^+^. “New” means it has been reported from *E. africanus* for the first time, although it has been previously identified from other plant sources, as referenced in the main text.

**Table 4:** Linearity, LOD and LOQ of the chemical markers used as reference.

No	Name	t_R_ (min)	UV_λmax_ (nm)	Regression Equation	Linear Range mg/L	R^2^	LOD * mg/L	LOQ * mg/L
1	Neochlorogenic acid	7.1	300, 325	y = 3.0x + 7.9	3.9–31.3	0.994	10.0	30.4
2	Catechin	8.7	278	y = 1.6x + 94.2	3.9–31.3	0.999	5.3	16.0
3	Caffeic acid	9.4	300, 325	y =1.9x − 1497.4	3.9–15.6	0.993	6.2	18.9
4	Epicatechin	10.8	277	y = 1.2x + 1859.3	3.9–31.3	0.996	4.0	12.2
5	p-Coumaric acid	11.8	300, 308	y = 3.9x + 2855.3	7.8–62.5	0.993	12.9	39.1
6	Ferulic acid	13.4	300, 323	y = 4.2x + 1098.7	7.8–31.3	1.000	13.8	41.7
7	Rutin	14.5	254, 255, 354	y = 1.9x + 449.7.3	7.8–31.3	0.994	6.1	18.6
8	Phloridzin	17.9	284	y = 1.4x + 1603.3	3.9–15.6	1.000	4.7	14.2

* Limit of detection (LOD), Limit of quantification (LOQ).

## Data Availability

Not applicable.

## Sample Availability

Samples of the compounds are available from the authors on request.
